# Paradoxical Thalamic Infarction via Patent Foramen Ovale in an Athletic Young Adult: A Case Report

**DOI:** 10.7759/cureus.91620

**Published:** 2025-09-04

**Authors:** Mohammed H Elaghory, Mohammad Bayer

**Affiliations:** 1 General Medicine, Glangwili General Hospital, Carmarthen, GBR

**Keywords:** cryptogenic stroke, patent foramen ovale (pfo), pfo, thalamic infarct, young adult

## Abstract

Cryptogenic ischemic stroke represents a significant diagnostic challenge in young adults. A patent foramen ovale (PFO) is an increasingly recognized potential etiology, facilitating paradoxical embolism. We report the case of an 18-year-old athletic female who presented with acute neurological symptoms consistent with a right thalamic ischemic stroke, confirmed on magnetic resonance imaging (MRI). Extensive workup excluded common stroke causes, and the case fulfilled Embolic Stroke of Undetermined Source (ESUS) criteria. A PFO was identified via contrast transthoracic and transesophageal echocardiography (TTE and TEE). The patient underwent successful percutaneous PFO closure approximately four weeks after symptom onset and achieved full neurological recovery. This case highlights PFO as a crucial consideration in the differential diagnosis of cryptogenic stroke in young, otherwise healthy individuals. It underscores the importance of comprehensive investigation, including contrast echocardiography, and supports PFO closure in appropriately selected patients, such as those under 60 years of age with non-lacunar cryptogenic stroke and confirmed PFO, consistent with current evidence.

## Introduction

Ischemic stroke, a major cause of mortality and long-term disability worldwide, is increasingly recognized as a significant health concern in young adults, often defined in the literature as individuals under 55 years of age [1]. Stroke in this population accounts for approximately 10-15% of all ischemic stroke cases, according to epidemiological data from multiple population-based studies [2]. The etiological landscape of stroke in the young differs considerably from that in older populations, encompassing a broader spectrum of causes, including large artery atherosclerosis, arterial dissection, cardioembolism, hypercoagulable states, inflammatory vasculopathies, and other less common conditions. Despite extensive diagnostic evaluations, a substantial proportion of ischemic strokes in young adults, estimated between 20% and 40%, remain cryptogenic, meaning no identifiable cause is found [2].

Standard cryptogenic stroke evaluation typically includes neurovascular imaging to exclude large artery disease, cardiac rhythm monitoring (e.g., Holter) to detect arrhythmias, echocardiographic assessment for structural or embolic cardiac sources, and laboratory testing for thrombophilic and autoimmune/inflammatory conditions [2-4]. In cases where a complete evaluation fails to reveal a clear cause, the stroke is often classified under the Embolic Stroke of Undetermined Source (ESUS) framework [3].

One increasingly recognized etiology in this context is paradoxical embolism through a patent foramen ovale (PFO). The foramen ovale is a physiological component of fetal circulation, which typically undergoes functional and anatomical closure after birth. Incomplete fusion results in a PFO, persisting in approximately 25% of the general adult population, with prevalence slightly declining with age [3,4]. While usually asymptomatic, a PFO can act as a conduit for venous thrombi to bypass the pulmonary circulation and enter systemic arterial circulation, thereby precipitating paradoxical embolic events such as stroke [1,3,4].

The association between PFO and cryptogenic stroke has been demonstrated by numerous independent case-control and cohort studies and further reinforced by systematic reviews and meta-analyses [1,4-6]. For example, a 2018 meta-analysis by Mojadidi et al. reported a pooled odds ratio (OR) of 3.1 (95% CI: 2.3-4.0) for the presence of a PFO in patients with cryptogenic stroke compared to controls [4]. This association is particularly strong in younger patients without conventional vascular risk factors. The prevalence of PFO in patients under 55 with cryptogenic stroke is estimated at 40-50%, compared to 10-25% in those with stroke of known cause [1,4,7].

Recent randomized controlled trials (RCTs) such as RESPECT, CLOSE, and REDUCE have demonstrated that percutaneous PFO closure significantly reduces the risk of recurrent stroke in carefully selected patients [5,6,8]. These trials defined high-risk PFOs as those with large right-to-left shunts (typically >20-30 microbubbles observed on contrast imaging), the presence of an atrial septal aneurysm (ASA), or both. Patients under 60 years of age with cryptogenic non-lacunar stroke, absence of alternative etiology, and confirmation of PFO by contrast echocardiography were considered ideal candidates. While our patient was only 18 years old-somewhat younger than the median age in these trials-she met all other major inclusion criteria. Her case aligns with existing clinical precedent for closure in adolescents, supported by shared pathophysiological mechanisms and favorable safety data [1,4,9].

Although paradoxical embolism more commonly causes cortical infarcts, deep infarcts such as those in the thalamus have also been reported, depending on embolus size and cerebral arterial anatomy [4,9]. Recognizing such variability in infarct localization is important when evaluating potential PFO-related strokes.

We present the case of an 18-year-old, highly athletic woman who experienced an acute ischemic stroke localized to the right thalamus. Comprehensive investigation excluded alternative stroke etiologies, and contrast-enhanced echocardiography confirmed the presence of a PFO. This case underscores the relevance of PFO in very young adults with cryptogenic stroke and illustrates the successful application of contemporary diagnostic and therapeutic strategies, including PFO closure.

## Case presentation

An 18-year-old previously healthy, highly athletic woman presented with sudden-onset neurological symptoms. The initial neurological symptoms included sudden light-headedness, unsteadiness, a transient left facial droop observed by her parents, and a subjective feeling of reduced control over her left arm. These symptoms showed gradual improvement shortly after onset. Two to three days later, she developed right periorbital headaches associated with photophobia and fatigue, but without nausea. These resolved with paracetamol. Although the symptoms were initially considered possibly migrainous, the absence of migraine history and the presence of focal neurological signs raised concern for a vascular event.

She denied any history of previous neurological symptoms, seizures, or transient ischemic attacks. There was no history of easy bruising, mucosal bleeding, or heavy menstrual bleeding. She was not taking any medications, including oral contraceptives or over-the-counter supplements. There was no recent immobilization, travel, surgery, or malignancy. The patient had no known arrhythmias, palpitations, or cardiac disease. Her family history was negative for stroke, thrombophilia, or bleeding disorders.

The patient presented for medical attention approximately five days after symptom onset. Her initial neurological symptoms, including facial droop and left arm weakness, had largely resolved within 24 hours of onset. On examination at presentation, she was alert and oriented with normal speech. Cranial nerves, including visual fields, were intact, with no residual facial weakness. Motor examination revealed normal tone and full power (5/5) throughout all limbs. Reflexes were symmetrical, and plantar responses were downgoing. Sensory and coordination testing, including assessment for ataxia, were also normal.

**Figure 1:**
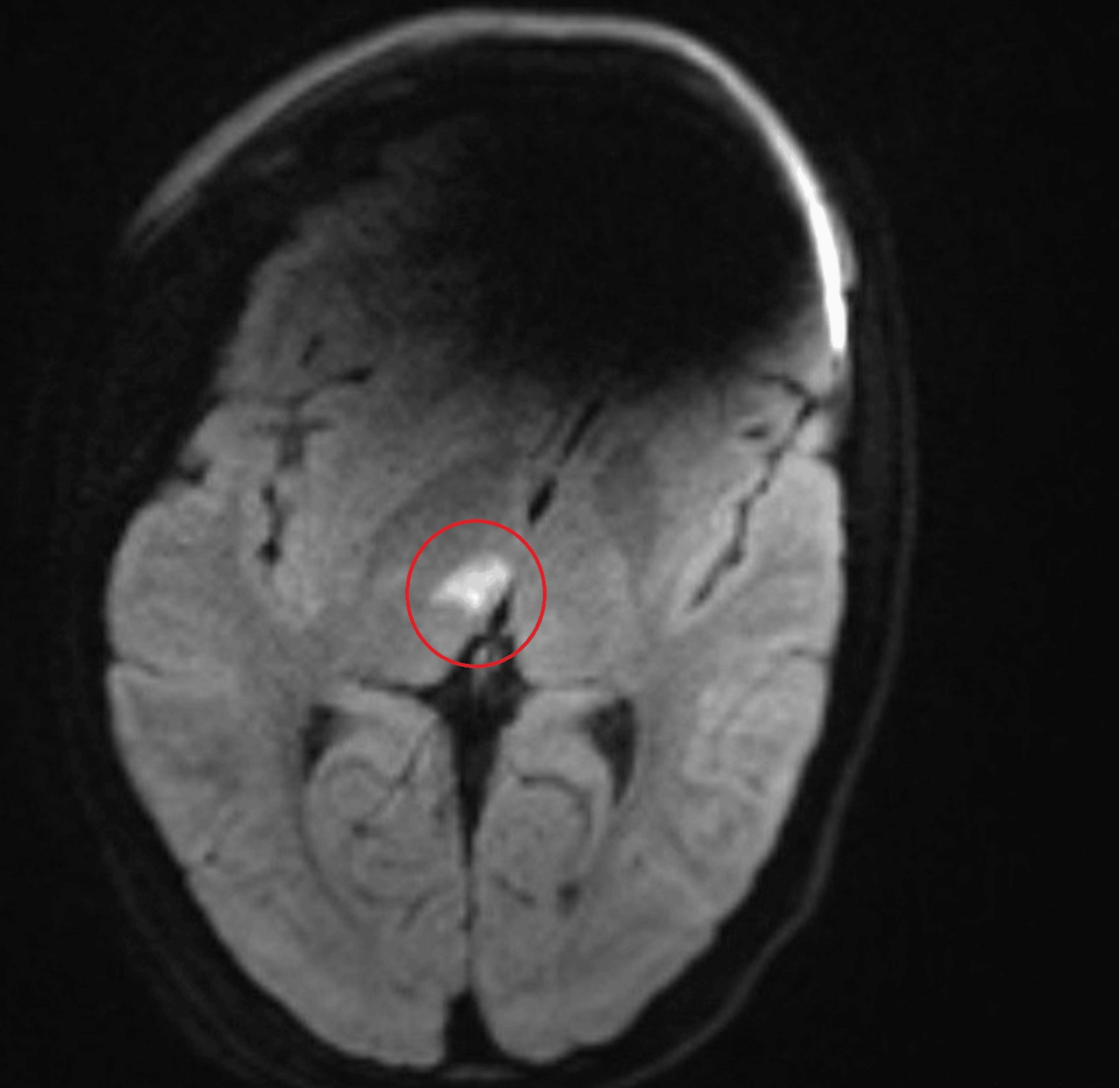
MRI brain, DWI sequence, showing an area of restricted diffusion in the right thalamus, consistent with an acute ischemic infarct (outlined in red).

Initial diagnostic imaging included a non-contrast computed tomography (CT) scan of the head, performed to exclude intracranial hemorrhage as per standard acute stroke protocols. Although the patient presented five days after symptom onset, no definite ischemic lesion was visible on CT. A CT angiogram of the head and neck showed no large vessel occlusion. Given the patient’s young age, atypical thalamic lesion location, and absence of conventional vascular risk factors, magnetic resonance venography (MRV) was performed to exclude cerebral venous sinus thrombosis. The MRV outlined a small focal lesion (~1 cm) within the right thalamus, but interpretation was limited as diffusion-weighted imaging (DWI) was not obtained in that sequence. A subsequent MRI of the brain with DWI, performed the same week, confirmed the presence of an acute right thalamic infarct (Figure 1)..

Notably, the patient had sustained a minor right knee injury seven days prior to the onset of neurological symptoms, which caused localized swelling but did not lead to immobility, require medical treatment, or limit her daily activities. Given this context, the possibility of deep vein thrombosis (DVT) was considered; however, clinical examination showed no signs of active thrombosis, and Doppler ultrasound was not performed due to low suspicion.

Laboratory investigations included a full hypercoagulable and autoimmune workup, all of which were negative or within normal limits: IgM and IgG anticardiolipin antibodies, anti-beta-2-glycoprotein I antibodies, antinuclear antibody screen, anti-dsDNA antibodies, immunoglobulin levels (IgG, IgA, IgM), complement levels (C3, C4), rheumatoid factor, protein C activity assay, and lupus anticoagulant screening. PT, APTT, and fibrinogen levels were normal. Alpha-glucosidase levels were also normal.

**Figure 2:**
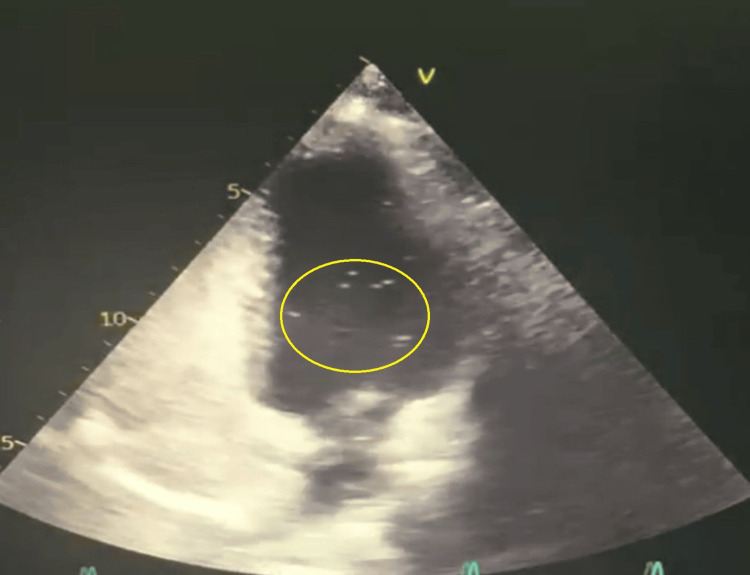
Bubble-contrast transthoracic echocardiogram (TTE) demonstrating an intracardiac shunt. Microbubbles are visible in the left atrium shortly after right atrial opacification, consistent with right-to-left shunting. The positive findings are highlighted with a yellow circle.

**Figure 3:**
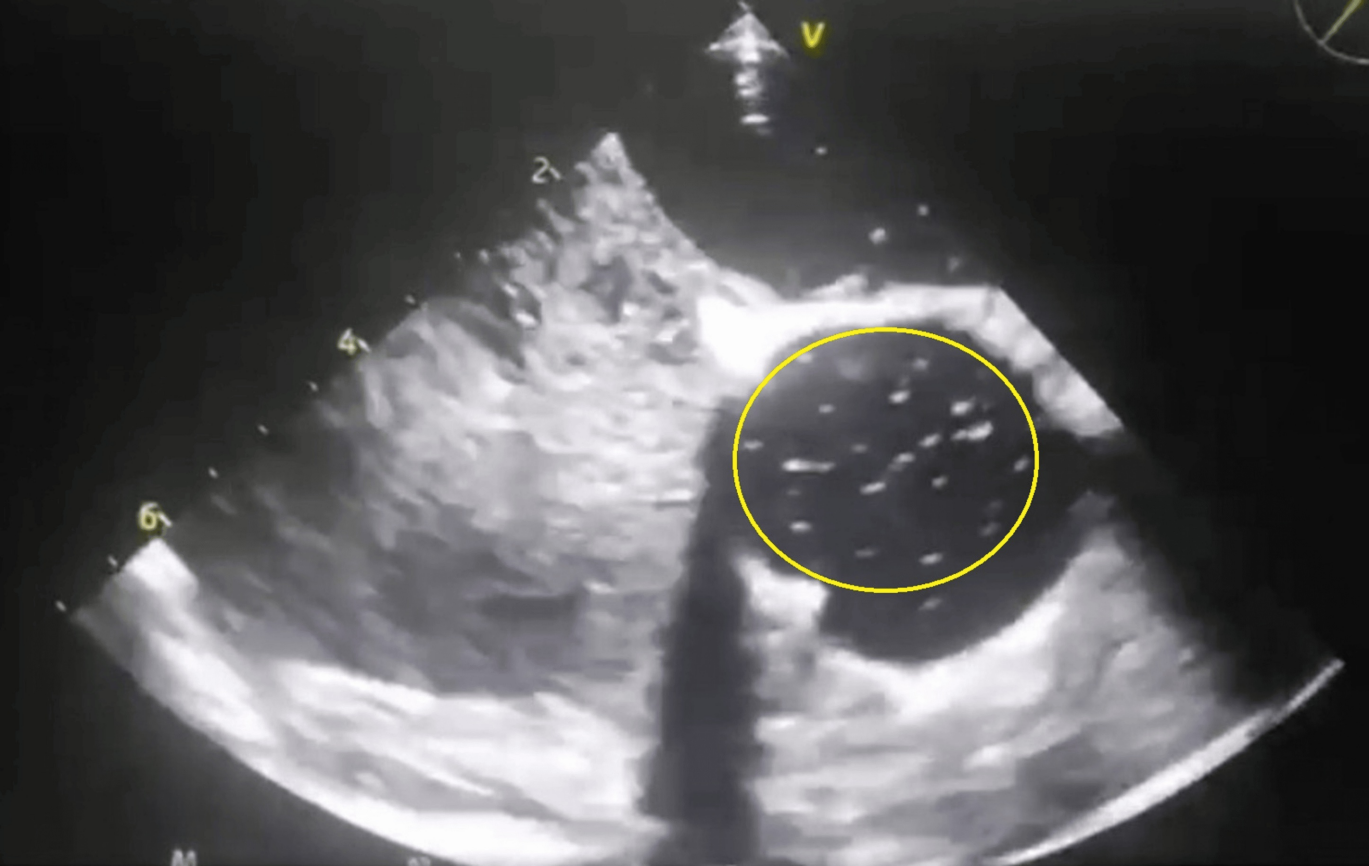
Bubble-contrast transesophageal echocardiogram (TEE) confirming the presence of a patent foramen ovale (PFO). More than 20 microbubbles are seen crossing from the right atrium to the left atrium within three cardiac cycles, consistent with a large right-to-left shunt. The study was performed with a Valsalva maneuver to accentuate shunting. No precise measurements of tunnel length or septal mobility were obtained, which is acknowledged as a limitation. The area of shunting is highlighted with a yellow circle.

Cardiac evaluation began with a transthoracic echocardiogram (TTE), which revealed no structural abnormalities. A contrast bubble TTE demonstrated an intracardiac right-to-left shunt (Figure 2). Transesophageal echocardiography (TEE) confirmed a large right-to-left intracardiac shunt (>20 microbubbles entering the left atrium within three cardiac cycles during Valsalva, consistent with a Grade 3 shunt) (Figure 3). No atrial septal aneurysm (ASA) was identified, and there was no spontaneous right-to-left shunting at rest. A possible aortic fibroelastoma was considered but ruled out by cardiac MRI, which showed no cardiac masses and normal biventricular function. CT coronary angiography revealed normal coronary arteries. Prolonged rhythm monitoring for 48 hours using Holter ECG revealed no arrhythmias.

The patient’s case was discussed in a multidisciplinary PFO team meeting. In the absence of other stroke etiologies and with the presence of a high RoPE score (estimated 9-10), a large PFO, and young age, the decision was made to proceed with percutaneous PFO closure. She was started on antiplatelet therapy following the diagnosis of ischemic stroke. She subsequently underwent successful transcatheter closure of the PFO approximately four weeks after the initial presentation at a specialized center. Detailed peri- and postprocedural pharmacologic management was not available. A follow-up echocardiogram confirmed that the closure device was well seated without complications.

## Discussion

This report details the case of an acute ischemic stroke involving the right thalamus in an 18-year-old, otherwise healthy and athletic woman. The diagnosis of ischemic stroke was confirmed by MRI demonstrating restricted diffusion in the right thalamus, a finding consistent with acute infarction. The subsequent comprehensive etiological workup systematically excluded common causes of stroke in young adults [1]. Neurovascular imaging (CTA/MRA) showed no evidence of large vessel occlusion, dissection, or significant atherosclerosis. Cardiac investigations, including 48-hour Holter monitoring and structural assessment via TTE and cardiac MRI, ruled out significant structural heart disease, valve abnormalities, or intracardiac masses such as the initially suspected fibroelastoma. Furthermore, extensive laboratory testing for hypercoagulable states (including antiphospholipid syndrome, protein C deficiency, and others) and relevant autoimmune/inflammatory conditions yielded negative results.

In this context of an otherwise cryptogenic stroke, the definitive finding of an intracardiac shunt, identified by both contrast-enhanced TTE and TEE and consistent with a PFO, becomes highly significant. Paradoxical embolism via a PFO is a well-established mechanism for stroke, particularly in younger individuals lacking traditional vascular risk factors [1,3,4]. TEE confirmed a large shunt (>20 bubbles with Valsalva, Grade 3), but no atrial septal aneurysm (ASA) or spontaneous right-to-left shunting at rest was observed. Other anatomical features, such as tunnel length, were not systematically assessed and represent a limitation in fully characterizing the PFO.

Assessing the likelihood of the PFO being pathogenic versus incidental is crucial. The Risk of Paradoxical Embolism (RoPE) score is a validated tool that estimates this probability based on patient age, infarct characteristics (cortical vs. non-cortical), and the absence of traditional vascular risk factors [3]. For this 18-year-old patient with a subcortical (thalamic) infarct and no reported hypertension, diabetes, smoking history, or prior stroke/TIA, the RoPE score would likely be high (potentially 9 or 10). However, it is important to note that the model was primarily validated in cortical strokes, and its application to subcortical events such as thalamic infarction may be less robust.

An interesting aspect of this case is the history of a minor knee injury with swelling preceding the neurological event. Such an event raises the possibility of transient venous thrombus formation that could have served as the embolic source. However, Doppler ultrasound was not performed, representing a diagnostic gap. Alternatively, recent literature suggests that trigger factors such as vigorous exercise, fever, or preceding flu-like illness might increase the risk of PFO-related stroke [9], potentially by transiently increasing right atrial pressure or promoting a prothrombotic state. The patient’s athletic background might also involve activities predisposing to Valsalva maneuvers, which can facilitate right-to-left shunting.

Alternative explanations for the MRI lesion, such as demyelination or migraine-related infarct-like lesions, were also considered. However, restricted diffusion with ADC correlate, absence of prior neurological history, and the clinical presentation supported an ischemic etiology, strengthening diagnostic confidence.

Given the diagnosis of a PFO-associated cryptogenic stroke in a young patient, the decision to proceed with PFO closure aligns with current evidence and guidelines. The patient was started on antiplatelet therapy after the diagnosis of stroke and subsequently underwent closure at a specialized center, although peri- and postprocedural pharmacologic management details were not available. Multiple large RCTs (RESPECT [8], CLOSE, REDUCE) and subsequent meta-analyses [5,6] have demonstrated that percutaneous PFO closure is superior to medical therapy alone in reducing the risk of recurrent stroke in carefully selected patients, particularly those under 60 years with cryptogenic stroke and high-risk PFO features. These studies demonstrated relative risk reductions of approximately 50-70%, with absolute risk reductions of 2-4% over follow-up periods of several years. The patient’s excellent outcome following the procedure is consistent with the generally favorable safety profile of contemporary PFO closure techniques [5,6,10].

While PFO closure was successful in this patient, the incomplete characterization of certain PFO risk features (such as tunnel length and septal mobility) represents a limitation and may affect the generalizability of conclusions. Referral to specialized centers is particularly warranted when cryptogenic stroke is confirmed after standard etiologic workup and a PFO is identified with high-probability features, including young age, absence of vascular risk factors, high RoPE score, cortical infarct patterns, or echocardiographic evidence of large shunt or ASA. Equally important is structured follow-up to monitor the closure device, ensure adherence to secondary stroke prevention strategies, and allow timely reassessment should new risk factors emerge.

It is also worth noting that more recent randomized studies and pooled analyses have since been published, further reinforcing the benefit of closure in high-risk patients, although these were not included in the present review.

## Conclusions

This case illustrates the importance of considering a PFO as a potential etiology in young adults presenting with cryptogenic stroke, even in the absence of traditional vascular risk factors. It underscores the value of a systematic diagnostic approach, including contrast echocardiography, in identifying this potentially modifiable stroke risk factor. The successful outcome following PFO closure reinforces the efficacy of this intervention in carefully selected patients, as supported by contemporary evidence. At the same time, the absence of detailed PFO anatomical characterization in this case limits generalizability, and we acknowledge this as a reporting limitation. The case also highlights the potential educational value of evaluating possible provoking factors, such as recent trauma with swelling, though this remains a hypothesis-generating observation rather than an established mechanism. Clinicians should maintain a high index of suspicion for PFO in young stroke patients and consider referral to specialized centers with multidisciplinary expertise in PFO evaluation, closure, and follow-up care.
